# Familial Mediterranean Fever: Recent Developments in Pathogenesis and New Recommendations for Management

**DOI:** 10.3389/fimmu.2017.00253

**Published:** 2017-03-23

**Authors:** Seza Özen, Ezgi Deniz Batu, Selcan Demir

**Affiliations:** ^1^Department of Pediatrics, Division of Rheumatology, Hacettepe University Faculty of Medicine, Ankara, Turkey

**Keywords:** familial Mediterranean fever, *MEFV*, pyrin, colchicine, Rho GTPases

## Abstract

Familial Mediterranean fever (FMF) is the most common monogenic autoinflammatory disease (AID) affecting mainly the ethnic groups originating from Mediterranean basin. The disease is characterized by self-limited inflammatory attacks of fever and polyserositis along with elevated acute phase reactants. FMF is inherited autosomal recessively; however, a significant proportion of heterozygotes also express the phenotype. FMF is caused by mutations in the *MEFV* gene coding for pyrin, which is a component of inflammasome functioning in inflammatory response and production of interleukin-1β (IL-1β). Recent studies have shown that pyrin recognizes bacterial modifications in Rho GTPases, which results in inflammasome activation and increase in IL-1β. Pyrin does not directly recognize Rho modification but probably affected by Rho effector kinase, which is a downstream event in the actin cytoskeleton pathway. Recently, an international group of experts has published the recommendations for the management of FMF. Colchicine is the mainstay of FMF treatment, and its regular use prevents attacks and controls subclinical inflammation in the majority of patients. Furthermore, it decreases the long-term risk of amyloidosis. However, a minority of FMF patients fail to response or tolerate colchicine treatment. Anti-interleukin-1 drugs could be considered in these patients. One should keep in mind the possibility of non-compliance in colchicine-non-responders. Although FMF is a relatively well-described AID and almost 20 years has passed since the discovery of the *MEFV* gene, there are still a number of unsolved problems about it such as the exact mechanism of the disease, symptomatic heterozygotes and their treatment, and the optimal management of colchicine resistance.

## Introduction

Familial Mediterranean fever (FMF) is the most common monogenic autoinflammatory disease (AID) over the world. Its prevalence is very high among people from the eastern Mediterranean such as Jews, Turks, Armenians, and Arabs (1, 2). However, patients from different ethnicities (such as Japan) are being increasingly recognized (3, 4). Self-limited inflammatory attacks of fever and polyserositis along with high acute phase response are the typical phenotype expected in FMF (5). The most significant complication of FMF is amyloidosis, and it is responsible for long-term morbidity and mortality (6). Although it is known to be inherited autosomal recessively, a substantial number of heterozygotes are present expressing the phenotypic characteristics (7).

Since the definition of *MEFV* gene mutations underlying FMF in 1997, around 310 sequence variants in *MEFV* gene have been detected (8). *MEFV* gene, located on chromosome 16 encodes for pyrin protein (9, 10). Pyrin, exists mostly in neutrophils and macrophages, has a key role in apoptosis and inflammatory pathways (9, 11). Mutated pyrin causes an exaggerated inflammatory response by uncontrolled interleukin-1 (IL-1) secretion (11). Recent studies have supplied information about the importance of the role of pyrin as a pattern recognition receptor (PRR), as well (12).

Colchicine is the mainstay of FMF treatment, and its regular use prevents attacks and suppresses chronic subclinical inflammation (13–15). Anti-IL-1 drugs emerged as promising treatment options in patients who fail to response or tolerate colchicine. Compliance to this orally administered drug is a problem. In resistant cases, the clinicians should also keep in mind whether the patient is compliant to the therapy (16). Recently, a group of international experts has published the recommendations for the management of FMF to guide physicians taking care of these patients (17).

In this review, we will discuss the new findings in the pathogenesis of FMF and the new recommendations for management.

## Genetics of FMF

In 1997, mutations in the *MEFV* gene, composed of 10 exons and located on chromosome 16 (16p13.3), were found to be associated with FMF (9, 10, 18). The gene encodes a 781 amino acid protein termed pyrin or marenostrin (9, 10, 18). Only a few mutations had been defined in selected families when the genetic association was first described (10, 19). Up to date, according to the INFEVERS database, more than 310 *MEFV* sequence variants have been reported (http://fmf.igh.cnrs.fr/infevers/). However, all variants are not associated with a disease phenotype and are termed “variants of uncertain significance.” With the description of new mutations, concerns emerged for the adequacy of checking only the common mutations. Booty et al. sequenced the *MEFV* gene in FMF patients and showed that screening the most common mutations instead of sequencing the whole gene appears sufficient to diagnose FMF in presence of clinical symptoms (20). In 2012, a group of clinical and molecular experts reached a consensus to test for a total of 14 *MEFV* variants if possible (21). These include nine clearly pathogenic variants (M694V, M694I, M680I, V726A, R761H, A744S, I692del, E167D, and T267I) and five variants of unknown significance (E148Q, K695R, P369S, F479L, and I591T) (21).

In the Eastern Mediterranean, the distribution of *MEFV* mutations is quite similar. M694V is the most common mutation in Turk (5), Armenian (22, 23), Arab (24), and Jewish populations (25); however, it is less common in Arabs (26). The second most common mutation is M680I in Turks (5); and V726A in Armenians (22, 23), Arabs (24), and Jews (25). M680I is the third most common mutation in Armenians (23). M694I is mostly seen in the Arabic population (24). On the other hand, in populations where FMF is a rare disease, the aforementioned mutations are less common, and other mutations are also seen. For example, in Japanese patients, E148Q is the most common variant followed by M694I and L110P (3). The clinical variability in FMF could be partly explained by genetic heterogeneity. For instance, most experts agreed that M694V was associated with a severe disease phenotype (8).

Recently, evidence-based recommendations have been developed for genetic diagnosis of FMF by the Single Hub and Access point for pediatric Rheumatology in Europe (SHARE) initiative (8). These recommendations are presented in Table 1. According to these, patients homozygous for M694V should be considered at higher risk of early disease onset and developing a severe phenotype (8). Furthermore, the patients carrying two mutated alleles in position 680–694 on exon 10 are also considered at risk of having a more severe disease (8).

**Table 1:** **Recommendations for familial Mediterranean fever (FMF) genetic diagnosis [adapted from Ref. (8)]**.

Recommendation	Strength of evidence
1. FMF is a clinical diagnosis, which can be supported but not excluded by genetic testing	B
2. Consider patients homozygous for M694V at risk of developing, with very high probability, a severe phenotype	B
3. FMF patients carrying two of the common mutated alleles (homozygotes or compound heterozygotes), especially for M694V mutation or mutations at position 680–694 on exon 10, must be considered at risk of having a more severe disease	B
4. The E148Q variant is common, of unknown pathogenic significance, and as the only *MEFV* variant does not support the diagnosis of FMF	B
5. Patients homozygous for M694V mutation are at risk of early onset disease	C
6. Individuals homozygous for M694V who are not reporting symptoms should be evaluated and followed closely in order to consider therapy	A
7. For individuals with two pathogenic mutations for FMF who do not report symptoms, if there are risk factors for AA amyloidosis (such as the country, family history, and persistently elevated inflammatory markers, particularly serum amyloid A protein), close follow-up should be started and treatment considered	B
8. Consultation with an autoinflammatory disease specialist may be helpful in order to aid in the indication and interpretation of the genetic testing and diagnosis	C

Another area of debate is E148Q variant. E148Q, the most frequent sequence alteration in the *MEFV* gene (27), is the result of the substitution of glutamine for glutamic acid at codon 148 in exon 2 (28, 29). E148Q is a common variant in the general population; however, the pathogenic role of E148Q is still uncertain (30).

In 2000, in a case–control study, Ben-Chetrit et al. found a similar frequency for E148Q mutation both in patients and healthy controls and in patients and their asymptomatic relatives (27). Tchernitchko et al. also demonstrated that E148Q allele frequency was comparable among patients and asymptomatic relatives and they concluded E148Q as a benign polymorphism (31). However, other studies (32, 33) showed that patients with homozygous E148Q variant might have an FMF-like phenotype requiring colchicine treatment. In a recent study, it has been suggested that the disease was less severe, the disease onset was later, and the ratio of patients responding completely to colchicine was higher in—at least a portion of—patients homozygous for E148Q when compared to the patients with exon 10 mutations (34).

Shinar et al. defined E148Q as a variant of unknown significance (21) and according to the SHARE recommendations, E148Q, as the only *MEFV* variant, does not support the diagnosis of FMF (8).

Although FMF is considered as an autosomal recessive disease, it was recognized that a significant portion of the patients had only one mutation in the *MEFV* gene (25, 35). Marek-Yagel et al. examined heterozygote FMF patients and performed haplotype studies in FMF families (36). They concluded that in some cases, the disease in heterozygotes could not be distinguished from that of homozygous patients, and FMF could be viewed as a dominant condition with low penetrance. Booty et al. searched for a second *MEFV* mutation in heterozygote patients who had a clinical diagnosis of FMF (20). However, re-sequencing the entire *MEFV* gene did not yield a second mutation in any of these cases (20). A recent study demonstrated that the frequency of FMF-like symptoms increased from patients carrying a single low penetrance mutation toward patients with two high-penetrance mutations suggesting a “dose effect” associated with mutations (37). One other explanation for heterozygote FMF patients may be the effect of the other modifier genes such as serum amyloid A (SAA) gene. *SAA* polymorphisms have been shown to contribute the severity of FMF phenotype inducing the expression of pro-interleukin-1β (IL-1β) and activating NLRP3 inflammasome resulting in the secretion of active IL-1β (4, 38). In the same lines, recently, Atoyan et al. have shown that SAA1 α allele was strongly associated with amyloidosis in FMF patients (39). On the other hand, environmental factors also have effect on the disease phenotype. Touitou et al. examined the characteristics of 2,482 FMF patients (260 of whom had amyloidosis) from 14 different countries (40). They demonstrated that the country of recruitment (roughly the same as the country of residence) was the most important determinant of amyloidosis risk.

We had shown that Turkish children with FMF in Germany expressed a less severe disease phenotype in comparison with the ones living in eastern Mediterranean (41). In addition, when we examined the Eurofever registry, we have seen that patients with a European ancestry have a milder disease than the Eastern Mediterranean patients (41). Furthermore, patients living in eastern Mediterranean countries had a higher frequency of fever episodes per year, and more frequent arthritis, pericarditis, chest pain, abdominal pain, and vomiting compared to the patients living in Western Europe (41). It was noteworthy that Western European patients had less frequent abdominal pain, pericarditis, and arthritis than eastern Mediterraneans (41). All of the above studies suggest the effect of environment on the phenotype of this monogenic disease.

Another issue that deserves a mention is autosomal dominant FMF. Different mutations (H478Y, T577S, T577A, T577N, M694del, M694I, E148Q, and L110P) in *MEFV* have been reported to cause dominant FMF in patients from different populations; Spanish, Turkish, Dutch, British, Indian, and Japanese (18, 41–45). These reports have shown that FMF could also be inherited autosomal dominantly, and these patients may have different disease phenotypes. Rowczenio et al. reported that symptoms may develop later in life in autosomal dominant FMF with p.M694Vdel than in classical recessive FMF (45). Stoffels et al. have demonstrated that these patients may have different symptoms during attacks such as urticarial rash and conjunctivitis overlapping with other AID (18).

Although this is a monogenic disease, epigenetic factors and microbiota may play role in the pathogenesis of FMF or phenotypic expression. It is tempting to speculate that host–microbe interactions may be important in this innate immune system disease. Khachatryan et al. demonstrated that the composition and divergence of microbiota were different during attack and attack-free periods as well as between FMF patients and healthy controls (46).

## Disease Pathogenesis

Pyrin, encoded by *MEFV*, has been suggested to interact with ASC (the inflammasome adaptor protein). The subsequent assembly of the inflammasome was suggested to activate caspase-1 leading to the cleavage and activation of IL-1β (47).

Until recently, it was a debate whether the disease-causing mutations in the *MEFV* gene were loss-of-function or gain-of-function mutations. There were different results depending on the different experimental settings. Supporting the loss-of-function model, Papin et al. demonstrated an increase in caspase-1 activation and IL-1β secretion as a result of pyrin knockdown (48). Hesker et al. showed that in response to inflammatory stimuli in a mouse line lacking the *MEFV* gene, IL-1β release by macrophages was enhanced (49).

On the other hand, in compliance with the gain-of-function model, Booty et al. demonstrated a significant increase in pyrin expression in FMF patients compared to healthy controls (20). Yu et al. have shown that activated pyrin forms a trimolecular complex by interacting with ASC and PTSPIP1, and this complex directly activates caspase-1 and leads to secretion of IL-1β (50). In 2011, Chae et al. have demonstrated that homozygous knock-in mice with the mouse pyrin protein fused to the human B30.2 domain containing FMF-associated mutations secrete large amounts of IL-1β in an NLRP3-independent manner (51). These data confirmed that the mutations associated with FMF were gain-of-function mutations and suggested that FMF was a pyrin inflammasomopathy (51).

Almost 20 years after defining the genetic basis of FMF and learning the role of pyrin in its pathogenesis, we now have some new data elaborating the role of pyrin in pathogenesis (12, 52). The detection of pathogenic microorganisms by PRRs triggers the formation of inflammasome (53). Recent data suggest that pyrin is also a PRR (12).

Two major virulence factors of *Clostridium difficile*, namely, TcdA and TcdB (54, 55) inactivate Rho GTPases *via* monoglycosylating a threonine residue in the GTPase switch I region of the protein (12). Recent studies have also shown that TcdB could trigger caspase-1 activation and IL-1β production; thus, it can activate the inflammasome (12, 56, 57). Furthermore, the C3 toxin of *Clostridium botulinum* and type VI secretion system (T6SS) of *Burkholderia cenocepacia* inactivate RHO by modifying the GTPase switch I region with different chemical groups and both trigger inflammasome activation (12). These results suggest that the bacterial toxins modifying RHO could trigger caspase-1 activation and IL-1β production; thus induce the inflammasome (12). The inflammasome activation by these toxins (TcdB, C3, and T6SS) was independent of NLRP3 and NLRC4 but was decreased in ASC^−/−^ and *MEFV^−/−^* bone marrow-derived macrophages (12). In addition, small interference RNA knockdown of pyrin inhibited TcdB-induced caspase-1 activation (12). These results also support the “gain-of-function” model in the pathogenesis of FMF. Since different inhibiting modifications in RHO proteins all result in caspase-1 activation, pyrin probably senses a downstream event in the actin cytoskeleton pathway (12).

The study by Park et al. has enlightened these mechanisms further (52). They have demonstrated that staurosporine, a potent inhibitor of protein kinase C that is an effector of RhoA, induced IL-1β release independent of the NLRP3, NLRC4, or AIM2 inflammasomes but dependent on the pyrin inflammasome (52). This shows that RhoA effector kinases suppress pyrin inflammasome activation. PKNs, RhoA effector kinases, bind to human pyrin and phosphorylate Ser208 and Ser242 units. The binding of PKN1 to the pyrin of FMF-knock-in mice (with B30.2 mutations; Mefv^M680I/M680I^, Mefv^M694V/M694V^, and Mefv^V726A/V726A^) was substantially decreased in comparison with the binding of PKN1 to wild type (Mefv^+/+^) mouse pyrin, which lacks a B30.2 orthologous domain (52). The binding of PKN1 to the pyrin of wild type B30.2 domain knock-in mice (Mefv^B30.2/B30.2^) was also decreased relative to wild-type mouse pyrin (but not as much as in FMF knock-in mice) (52). These suggest that the human B30.2 domain has a role in the regulation of PKN1 binding to pyrin. It was also shown that 14-3-3 protein binds to phospho-pyrin (phosphorylated from Ser208 and Ser242 units by PKNs) to inhibit inflammasome activation. Furthermore, the binding of 14-3-3 to mutant pyrin (M680I, M694V, and V726A) was decreased relative to wild-type human pyrin (52). All aforementioned results show that active RhoA signals through PKNs, which phosphorylate pyrin from Ser208 and Ser242 units. Then, 14-3-3 proteins bind to phospho-pyrin and inhibit the activation of pyrin inflammasome. Pyrin is activated when dephosphorylated at Ser208/Ser242. The binding of PKN1 to pyrin is decreased with the B30.2 domain where most of the common and severe *MEFV* mutations are clustered.

These data enlighten the effect of mutations on pyrin function and the downstream event of RhoA inhibiting pyrin. Active pyrin promotes ASC oligomerization and forms a caspase-1 activating complex resulting in IL-1β production. Wild-type pyrin relies selectively on microtubules for inflammasome activation and microtubules control pyrin signaling downstream of pyrin dephosphorylation (52). Recently, Van Gorp et al. have observed that colchicine pretreatment augments the TcdA-induced IL-1β secretion from FMF peripheral blood mononuclear cells (58). The microtubule assembly inhibition with nocodazole also had the same effect. Thus, FMF-associated mutated pyrin does not require microtubules for ASC speck assembly. *MEFV* mutations in B30.2 domain probably remove the critical reliance on intact microtubules for pyrin-based nucleation of ASC specks and inflammasome signaling (58).

To make the story even more complex, in a recent study, Kimura et al. have demonstrated that pyrin (referred as TRIM20 in the article) recognizes the inflammasome components, NLRP1, NLRP3, and procaspase-1 and leads to their autophagic degradation (59). Diminished autophagic degradation of NLRP3 was shown in single (M694V), double (M680I and M694V), and triple (M680I, M694V, and V726A) mutants (59).

When we look at the cellular level, we know that neutrophilia and influx of neutrophils to the inflamed sites occur in FMF attacks (60). Gohar et al. demonstrated that *in vitro*, unstimulated neutrophils from M694V positive patients spontaneously secreted more S100A12, IL-18, and caspase-1 compared to neutrophils from healthy controls (61). In another study, it has recently been shown that FMF attack is characterized by release of neutrophil extracellular traps (NET) including active IL-1β (60). These NET structures are observed in the first hours of FMF attacks, and subside as the inflammatory attack is resolved. They have demonstrated that NETs restrict their own generation by a negative feedback mechanism, which may be an explanation for the self-limited nature of FMF attacks. Of note, in this study, neutrophils from FMF patients in remission were resistant to induction of NET release. They have shown that reduced basal autophagy levels in these cells could be responsible for this since autophagy induction is needed for NET formation. Thus, lower basal autophagy levels of neutrophils may protect from attacks by attenuating the release of pro-inflammatory NETs.

Manukyan et al. have recently shown that the *ex vivo* spontaneous apoptotic rate of neutrophils from FMF patients in remission is significantly higher compared to control (62). The accelerated apoptosis of neutrophils in FMF may be important for successful resolution of inflammation and prevention of tissue damage. This may be another explanation for the self-limited nature of FMF attacks. Pyrin modulates the susceptibility to apoptosis; however, the effect of the mutant pyrin on apoptotic processes is poorly understood.

Although now we know more about the function of pyrin, the role of neutrophils, and the disease pathogenesis, there are still questions waiting to be answered such as the exact reason for the episodic and short-term nature of the inflammatory attacks and the phenotypic variability in FMF.

## Treatment

Familial Mediterranean fever can be well controlled with optimum standard management. Recently, with the international collaboration of experienced experts from different countries, the European League Against Rheumatism (EULAR) recommendation set for the management of FMF has been published supported by the best available evidence (17). These recommendations are presented in Table 2.

**Table 2:** **The European League Against Rheumatism recommendations for the management of FMF with grade of recommendation [adapted from Ref. (17)]**.

Recommendation	Grade
01. Ideally, FMF should be diagnosed and initially treated by a physician with experience in FMF	D
02. The ultimate goal of treatment in FMF is to reach complete control of unprovoked attacks and minimizing subclinical inflammation in between attacks	C
03. Treatment with colchicine should start as soon as a clinical diagnosis is made	A
04. Dosing can be in single or divided doses, depending on tolerance and compliance	D
05. The persistence of attacks or of subclinical inflammation represents an indication to increase the colchicine dose	C
06. Compliant patients not responding to the maximum tolerated dose of colchicine can be considered non-respondent or resistant; alternative biological treatments are indicated in these patients	B
07. FMF treatment needs to be intensified in AA amyloidosis using the maximal tolerated dose of colchicine and supplemented with biologics as required	C
08. Periods of physical or emotional stress can trigger FMF attacks, and it may be appropriate to increase the dose of colchicine temporarily	D
09. Response, toxicity, and compliance should be monitored every 6 months	D
10. Liver enzymes should be monitored regularly in patients with FMF treated with colchicine; if liver enzymes are elevated greater than twofold the upper limit of normal, colchicine should be reduced and the cause further investigated	D
11. In patients with decreased renal function, the risk of toxicity is very high, and therefore signs of colchicine toxicity, as well as CPK, should be carefully monitored and colchicine dose reduced accordingly	C
12. Colchicine toxicity is a serious complication and should be adequately suspected and prevented	C
13. When suspecting an attack, always consider other possible causes. During the attacks, continue the usual dose of colchicine and use NSAID	C
14. Colchicine should not be discontinued during conception, pregnancy, or lactation; current evidence does not justify amniocentesis	C
15. In general, men do not need to stop colchicine prior to conception; in the rare case of azoospermia or oligospermia proven to be related to colchicine, temporary dose reduction or discontinuation may be needed	C
16. Chronic arthritis in a patient with FMF might need additional medications, such as DMARDs, intra-articular steroid injections, or biologics	C
17. In protracted febrile myalgia, glucocorticoids lead to the resolution of symptoms; NSAID and IL-1-blockade might also be a treatment option; NSAIDs are suggested for the treatment of exertional leg pain	C
18. If a patient is stable with no attacks for more than 5 years and no elevated APR, dose reduction could be considered after expert consultation and with continued monitoring	D

*APR, acute phase reactants; CPK, creatinine phosphokinase; DMARDs, disease-modifying antirheumatic drugs; FMF, familial Mediterranean fever; IL-1, interleukin-1; NSAID, non-steroidal anti-inflammatory drugs*.

The EULAR recommendations emphasize that the aim of FMF treatment is obtaining the control of acute attacks, minimizing the chronic and subclinical inflammation, preventing complications, and providing an acceptable quality of life.

It is also emphasized that colchicine is the main treatment of FMF since 1972 (63). It is generally a safe and well-tolerated drug, but its mechanism of action in FMF has not been completely elucidated. However, we know that it prevents microtubule elongation by binding to tubulin monomers and inhibiting polymer formation (64, 65). Thus, the link between pyrin and colchicine could be through the organization of actin cytoskeleton.

Previously, it was claimed that colchicine is an activator of RhoA (66). It binds to tubulin, depolymerizes microtubules and causes release of the RhoA activator guanine-nucleotide-exchange factor-H1, which is inactive when bound to microtubules (66). Park et al. demonstrated that colchicine inhibited the constitutive IL-1β release from bone-marrow-derived macrophages (BMDMs) of Mefv^V726A/V726A^ mice and C3-toxin-induced IL-1β release from primed BMDMs. In addition, colchicine inhibited IL-1β release from PBMCs of FMF patients (52). In the same lines, Van Gorp et al. demonstrated that microtubule-depolymerizing drugs selectively inhibited the pyrin inflammasome (58). Thus, colchicine may be inhibiting pyrin inflammasome through RhoA activation by releasing RhoA activator from depolymerized microtubules.

Certain other pharmacological anti-inflammatory effects of colchicine have been enlightened such as preventing activation of neutrophils by forming β-tubulin–colchicine complexes and inhibiting the microtubule assembly and mitotic spindle formation, suppressing caspase-1 gene expression, and inhibiting the synthesis of tumor necrosis factor alpha (TNF-α) (65, 67–70).

It is suggested that colchicine should be started as soon as the patient is clinically diagnosed as having FMF. If the patient lacks clinical manifestations or subclinical inflammation, genetic diagnosis is not a precise indication to start treatment; however, these patients should be followed-up closely for clinical symptoms or signs of subclinical inflammation (17). In countries where amyloidosis has high frequency, the physician may consider treatment in these patients especially when the patient has homozygous M694V mutation, which is more frequently associated with the development of amyloidosis (9, 21, 71–77).

The optimal dosage of colchicine varies between studies and different clinical practices. The recommendation of the starting dose of colchicine in FMF is ≤0.5 mg/day for children <5 years of age; 0.5–1 mg/day for children 5–10 years of age; and 1–1.5 mg/day in children >10 years of age and in adults (in case tablet contains 0.6 mg; ≤0.6 mg/day; 1.2 mg/day; and 1.8 mg/day, respectively) (17). Higher starting doses could be used in patients with high disease activity or disease complications such as amyloidosis (17). However, in most patients, it is started at the subtherapeutic dose of 0.5 mg/day and adjusted according to disease activity and tolerance in the follow-up. While escalating colchicine dose in patients with active disease, monitoring C-reactive protein (CRP) and SAA, or both is required at least every 3 months (17). Both increase in attack frequency and presence of subclinical inflammation are indications to increase colchicine dose. The maximum dose is 2 mg/day in children and 3 mg/day in adults (14, 78). Dosing can be in single or divided doses. The dose can be divided to decrease side effects; however, a single daily dose may increase the compliance (17). Polat et al. have recently shown that using colchicine with either once- or twice-daily dosage provides similar clinical and laboratory improvement as well as the similar rate of drug side effect (79).

Colchicine treatment is lifelong in FMF. However, in EULAR recommendations, FMF experts recommend the consideration of colchicine dose reduction by an experienced center under certain circumstances with very careful and close follow-up (17).

Colchicine is a safe drug in the range of doses used for FMF treatment (80). The most common side effects of the drug and toxicity are also reviewed in the aforementioned recommendations. The most common side effect is gastrointestinal disturbance, which may be seen in up to 10% of patients during the first month of the treatment (81, 82). It was shown that jejunal lactase, sucrase, and maltase activities decreased in patients on long-term colchicine treatment (83). In these patients, increased fecal excretion of starch, fat, and bile acids and decreased absorption of d-xylose and vitamin B12 occur, as well. These may be the explanation for diarrhea and lactose intolerance, and a symptomatic relief can be provided with a lactose-free diet (83, 84). Dose reduction may also improve the gastrointestinal symptoms (85). There are also some rare side effects of colchicine, such as vitamin B12 deficiency, reversible peripheral neuritis and myopathy, bone marrow suppression, and alopecia (86–89). In addition, some animal studies and case reports suggested its association with azoospermia (90, 91); however, this was only in very high doses. Thus, in general, men need not stop colchicine prior to conception (17). Colchicine use is safe during pregnancy and lactation, as well (92–94). However, it should be used cautiously in patients with impaired renal or hepatic functions (95).

Compliance with colchicine is very important for proper management of FMF. One study showed that proteinuria that is usually the first sign of renal amyloidosis, developed after a period of 9–11 years in 1.7% of 960 adult patients who properly used colchicine versus 49% in 54 patients who were not compliant (96). There is a surprisingly high rate of incompliance with colchicine especially among adolescent patient (17). Thus, in the case of patients not responding to colchicine, the physician should keep in mind the possibility of incompliance. Overall, up to 5% of FMF patients may not respond to colchicine treatment and another 2–5% is colchicine intolerant (85).

Anti-IL-1 therapy seems to be a promising second-line therapy in refractory or intolerant patients. However, one should keep in mind that colchicine should be coadministered with biologic therapies since it may reduce the risk of amyloidosis (17). There are three types of anti-IL-1 agents in clinical use; anakinra, a recombinant homolog of the human IL-1 receptor (97); canakinumab, a fully human immunoglobulin G1 monoclonal antibody (98); and rilonacept, a dimeric Fc-fusion protein capturing IL-1 (97); all administered subcutaneously.

The most recent systematic review of the literature (99) has yielded 24 case reports/series, 2 open-label prospective trials (100, 101), and 1 placebo-controlled prospective trial (102) on anti-IL-1 use in FMF. Eighteen reports were on treatment with anakinra (103–120), four on canakinumab (100, 101, 121, 122), four on patients treated with either anakinra or canakinumab (123–126), and the only placebo-controlled prospective trial was on treatment with rilonacept (102). A complete response to therapy was reported in 76.5% of patients on anakinra, and 67.5% of patients on canakinumab treatment (99). In addition, IL-1 blockade can reverse proteinuria in patients with renal AA amyloidosis (99, 127). However, we do not know whether anti-IL-1 therapies could prevent amyloidosis. A new study on efficacy/safety of canakinumab in patients with hereditary periodic fevers including FMF is also underway (http://ClinicalTrials.gov identifier NCT02059291).

Anti-IL-1 drugs may be used “on demand” (starting at first symptom of attacks) in mevalonate kinase deficiency (128). We need further data on whether this would be an option for selected cases in FMF or on certain occasions.

Besides IL-1 blockade, FMF patients with chronic arthritis and/or sacroiliitis could benefit from disease-modifying antirheumatic drugs or anti-TNF agents (129, 130).

Treatment of protracted febrile myalgia syndrome (PFMS) has also been addressed. PFMS is a very rare manifestation of FMF and is defined as severe, disabling myalgia of at least 5 days duration (108, 112). It is associated with fever, the presence of at least one M694V mutation, and elevated inflammatory markers while creatine kinase levels are usually normal (131, 132). Corticosteroid treatment is required to suppress symptoms (17, 131, 133, 134). Non-steroidal anti-inflammatory drugs may also be beneficial (131). In addition, anakinra has been used successfully in two patients with PFMS associated with FMF (112).

### Treatment in Heterozygotes

Familial Mediterranean fever is a clinical diagnosis, and we have many patients who are heterozygous for *MEFV* mutations. How patients with one mutation only can express the disease is still not clear (135). We give colchicine treatment to patients who express the typical FMF phenotype. However, some heterozygotes can sometimes “outgrow” the phenotype (30). Ben-Zvi et al. previously demonstrated that their patients (not using colchicine) experienced years of symptom-free interval where 22 out of these 33 were heterozygotes (136).

The data on remission of the disease in heterozygotes are limited. Recently, we have reported our experience on heterozygote patients with transient FMF clinic (7). We discontinued colchicine treatment in 22 heterozygote FMF patients who had an inflammation- and attack-free period for a long duration. The median follow-up after colchicine cessation was 22.5 months, and we restarted colchicine in only two patients because of the recurrence of attacks. However, after colchicine cessation, close follow-up is crucial every 3–6 months to evaluate whether they have recurrence of attacks or subclinical inflammation.

### Refractory FMF and Outcome

There is no standard definition for refractory FMF patients. However, in the recent guideline, we stated that patients who continue to have ≥1 attacks per month despite receiving the maximally tolerated dose for ≥6 months might be considered non-responder or resistant to colchicine (17). Another issue is ongoing subclinical inflammation, which leaves the patients at risk of developing amyloidosis (17). In addition, in the case of AA amyloidosis, the FMF treatment should be intensified with biologics and maximal tolerated dose of colchicine (17).

There are mainly two tools to evaluate outcome and disease activity in FMF; FMF50 score and autoinflammatory disease activity index, respectively.

In FMF50, the items are percentage change in the frequency and duration of attacks, arthritis attacks, physician’s and patient’s/parents’ global assessment of disease severity (0–10 cm visual analog scale; 10 the worst), and in ESR, CRP, or SAA level with the treatment (137). At least 50% improvement in five out of six criteria by 3–6 months with no worsening in any one means FMF50 response. It is noteworthy that compliance with the maximum dose of drug is essential for evaluating the patients with FMF50 score.

Autoinflammatory disease activity index is a disease activity assessment tool for AID including FMF, and it is composed of 13 items: overall symptoms, nausea/vomiting, abdominal pain, diarrhea, chest pain, arthralgia or myalgia, swelling of the joints, headaches, eye manifestations, skin rash, and pain relief (138). Each item except pain relief is scored by the patients/parents for a total score of 0–34 in a single day and 0–1,054 in a month of 31 days. A cutoff score of ≥9 discriminates active from inactive patients with a sensitivity of 89% and specificity of 92% (138).

There is also one recent tool for AID including FMF to quantify damage in patients and to compare disease outcomes in clinical studies; autoinflammatory disease damage index (ADDI) (139).

In ADDI, damage is defined as “persistent or irreversible change in structure or function that is present for at least 6 months” (139). ADDI contains 18 items, and these items are categorized by organ systems as follows: reproductive, renal/amyloidosis, developmental, serosal, neurological, ears, ocular, and musculoskeletal. The renal/amyloidosis and neurological damage categories were assigned to have the highest number of points while serosal damage got the lowest. This index provides a universal instrument to measure damage by chronic inflammation in FMF.

These tools could aid us to form a standard definition for refractory FMF patients and standardize the outcome measurement in different studies.

## Unsolved Issues in FMF

As we mentioned above in the relevant parts, there are still gaps in knowledge about the pathogenesis and treatment mechanisms in FMF. We need further research on the following:

– the significance of the E148Q variant,
– exact roles of modifier factors (microbiota, microRNAs, etc.) on disease pathogenesis, phenotypic expression, and severity of the disease,
– the effects of mutant pyrin on apoptosis,
– the exact reason for the self-limited and episodic nature of disease attacks,
– whether anti-IL-1 treatment prevents amyloidosis,
– the definition of colchicine resistance,
– why certain rheumatic diseases are more common in heterozygotes, and why they sometimes express the disease phenotype,
– the duration of treatment in heterozygous patients,
– more biomarkers for secondary amyloidosis.

## Conclusion

When the mutated protein for FMF was described 20 years ago, we thought that everything was resolved. However, this monogenic disease continues to be of interest to clinical and basic researchers. We still need to address the above questions and the cause of the phenotypic heterogeneity in this disease. On the other hand, the experts on FMF have worked on compiling recommendations to guide physicians in the diagnosis, management, and treatment of FMF. It is hoped that these recommendations may be of practical use while the work on solving the pathogenesis continue.

## Author Contributions

EB and SD prepared the first draft of the article. SÖ made the critical revision of the article. All the authors have seen and approved the final version of the manuscript.

## Conflict of Interest Statement

SÖ has received consultancy and speaker fees from Novartis and Sobi and R-Pharma in the past. The remaining authors declare no conflict of interest.
