# Supplementary material for: Familial Mediterranean Fever: Recent Developments in Pathogenesis and New Recommendations for Management
Source: Front Immunol. 2017 Mar 23;8:253. doi: 10.3389/fimmu.2017.00253 (PMC5362626; doi:10.3389/fimmu.2017.00253)
Supplement: Supplementary file 1 [file Table_1.docx]

**Supplementary Table 1.** Final domains in the core set for the evaluation of response to treatment in familial Mediterranean fever (FMF): an FMF50 response is required which shows at least 50% improvement in at least ﬁve of these parameters with no worsening in one (adapted from reference no 137)

| **Outcome measures to deﬁne the response to treatment in FMF** |
| --- |
| 1. Percentage change in the frequency of attacks with the treatment |
| 2. Percentage change in the duration of attacks with the treatment |
| 3. Patients/parents’ global assessment of disease severity (10 cm VAS) |
| 4. Physicians’ global assessment of disease severity (10 cm VAS) |
| 5. Percentage change in arthritis attacks with the treatment |
| 6. Percentage change in CRP, ESR or SAA level with the treatment (at least 2 weeks after the last attack) |

CRP, C-reactive protein; ESR, erythrocyte sedimentation rate; FMF, familial Mediterranean fever; SAA, serum amyloid A; VAS, visual analogue scale.
