# Supplementary material for: Familial Mediterranean Fever: Recent Developments in Pathogenesis and New Recommendations for Management
Source: Front Immunol. 2017 Mar 23;8:253. doi: 10.3389/fimmu.2017.00253 (PMC5362626; doi:10.3389/fimmu.2017.00253)
Supplement: Supplementary file 2 [file Table_2.docx]

**Supplementary Table 2.** Auto-inflammatory disease activity index diary (adapted from reference no 138)

| Name: | | | | Age: | | | | Month: | | | | Year: | | | |
| --- | --- | --- | --- | --- | --- | --- | --- | --- | --- | --- | --- | --- | --- | --- | --- |
| Symptoms associated with auto-inflammatory syndrome today | | | | | | | | | | | | | | | |
| Day | Fever  ≥ 38 °C  (100.4°F) | Overall  symptoms | Abdominal  pain | | Nausea/  vomiting | Diar-rhoea | Head-  aches | | Chest  pain | Painful nodes | Arthralgia or myalgia | Swelling  of  the joints | Eye  manifestations | Skin rash | Pain  relief  drug  taken |
| Sco-re | 0 or 1 | 0 or 1 | 0 or 1 | | 0 or 1 | 0 or 1 | 0 or 1 | | 0 or 1 | 0 or 1 | 0 or 1 | 0 or 1 | 0 or 1 | 0 or 1 |  |
| 1 |  |  |  | |  |  |  | |  |  |  |  |  |  |  |
| 2 |  |  |  | |  |  |  | |  |  |  |  |  |  |  |
| 3 |  |  |  | |  |  |  | |  |  |  |  |  |  |  |
| ….. |  |  |  | |  |  |  | |  |  |  |  |  |  |  |
| 31 |  |  |  | |  |  |  | |  |  |  |  |  |  |  |

Each line refers to a day in a month
