# Supplementary material for: Familial Mediterranean Fever: Recent Developments in Pathogenesis and New Recommendations for Management
Source: Front Immunol. 2017 Mar 23;8:253. doi: 10.3389/fimmu.2017.00253 (PMC5362626; doi:10.3389/fimmu.2017.00253)
Supplement: Supplementary file 3 [file Table_3.docx]

**Supplementary Table 3.** Autoinflammatory Disease Damage Index (ADDI) including glossary of terms* (adapted from reference no 139)

| **Damage item** | **Grading** | **Points** |
| --- | --- | --- |
| **Reproductive** | - | Max. 3 |
| Sub/infertility | - | 2 |
| Amenorrhea | - | 1 |
| **Renal/amyloidosis** | - | Max. 6 |
| Amyloidosis | Limited amyloidosis | 2 |
|  | Extensive amyloidosis | 3 |
| Proteinuria | - | 1 |
| Renal insufficiency | Moderate renal insufficiency | 2 |
|  | Severe renal insufficiency | 3 |
| **Developmental** | - | Max. 3 |
| Growth failure | - | 2 |
| Puberty delay | - | 1 |
| **Serosal** | - | Max. 1 |
| Serosal scarring | - | 1 |
| Neurological | - | Max. 6 |
| **Developmental delay*** | - | 2 |
| Cognitive impairment | - | 3 |
| Elevated intracranial pressure | - | 2 |
| Central nervous system involvement | - | 3 |
| **Ears** | - | Max. 2 |
| Hearing loss | Moderate hearing loss of better ear | 1 |
|  | Severe hearing loss of better ear | 2 |
| **Ocular** |  | Max. 3 |
| Ocular involvement | Mild ocular involvement of better eye | 1 |
|  | Moderate ocular involvement of better eye | 2 |
|  | Severe ocular involvement of better eye | 3 |
| **Musculoskeletal** | - | Max. 4 |
| Joint restriction | - | 2 |
| Bone deformity | - | 2 |
| Osteoporosis | - | 1 |
| Musculoskeletal pain | - | 1 |

*Only for pediatric patients.
